# Association of Oncogenic Viral Infections With Cardiovascular Risk and Health Outcomes: A Cross-Sectional Study in South Asian Adults

**DOI:** 10.7759/cureus.95369

**Published:** 2025-10-25

**Authors:** Yashar Mashayekhi, Rabia Nazeer, Tasleem Tasleem, Shikha Tyagi, Mohammad Faiq Malik, Marvelous O Popoola, Thel Su Hlaing, Shehnaz Mahboob Ahmed, Muhammad Ibrar, Fahim Akhtar

**Affiliations:** 1 Medicine, University Hospitals of Leicester NHS Trust, Leicester, GBR; 2 General Medicine, D. G. Khan Medical College, Dera Ghazi Khan, PAK; 3 Critical Care, Luton and Dunstable University Hospital, Luton, GBR; 4 Internal Medicine, Bhagat Phool Singh (BPS) Government Medical College for Women, Khanpur Kalan, IND; 5 Emergency Department, Dr. Balasaheb Vikhe Patil Rural Medical College, Loni, IND; 6 Emergency, Calvary Adelaide Hospital, Adelaide, AUS; 7 Medicine, Nevill Hall Hospital, Abergavenny, GBR; 8 Family Medicine, Nad Al Hamar Health Center, Dubai, ARE; 9 College of Medicine, Mohammed Bin Rashid University of Medicine and Health Sciences, Dubai, ARE; 10 Internal Medicine, Combined Military Hospital (CMH) Lahore Medical College and Institute of Dentistry, Lahore, PAK

**Keywords:** cardiovascular risk, ihmrs, kccq, oncogenic viruses, south asia, vaccination, viral infections

## Abstract

Background: Cardiovascular disease (CVD) is a major global health issue. Chronic infections with oncogenic viruses such as human papillomavirus (HPV), hepatitis B virus (HBV), hepatitis C virus (HCV), Epstein-Barr virus (EBV), and human T-cell lymphotropic virus type 1 (HTLV-1) may increase cardiovascular risk. Evidence from high-burden regions, such as Pakistan and India, is limited. This study examines the association between oncogenic viral infections and cardiovascular health in South Asian adults.

Methods: This cross-sectional study was conducted in a community-based clinic in Pakistan and India from May to August 2025. The sample comprised 203 adults with a documented history of oncogenic viral infection (HPV, HBV, HCV, EBV, or HTLV-1) but without any previously diagnosed CVD. Structured questionnaires were used to collect data on demographic settings, viral infection, cardiovascular symptoms, and lifestyle factors. Cardiovascular health was assessed using the Kansas City Cardiomyopathy Questionnaire (KCCQ), and cardiovascular risk was evaluated using the INTERHEART Modifiable Risk Score (IHMRS). Statistical analysis was performed using the IBM SPSS Statistics for Windows, version 26 (IBM Corp., Armonk, New York, United States), which included descriptive statistics, correlation analysis, regression models, and the adjustment for confounding factors, including age, gender, and treatment history.

Results: Most of the participants were women (110, 54%), aged 36-45 years (60, 30%). A substantial positive relationship was found between the diagnosis of viral infection and the KCCQ (r = 0.298, p < 0.01) and IHMRS (r = 0.267, p < 0.01). Active viral disease was associated with decreased cardiovascular health outcomes in participants, as indicated by reduced KCCQ scores (p = 0.016). A significant gender difference was observed, with females exhibiting poorer cardiovascular health (p = 0.040). An association was observed between better cardiovascular outcomes and vaccination, timely treatment, and viral resorption.

Conclusion: The results suggest a significant association between oncogenic virus infections and cardiovascular health. Therefore, timely diagnosis, vaccination, and effective management are recommended to reduce cardiovascular risk. The findings underscore the importance of targeted health interventions, particularly in areas with high rates of viral infections, in mitigating long-term cardiovascular consequences.

## Introduction

Cardiovascular disease (CVD) is the leading cause of death in the world, accounting for approximately one-third, with limited preventive measures, especially in developing countries [1]. Globally, CVDs cause about 19.8 million deaths each year (32%), and viral infections like hepatitis B (HBV), hepatitis C (HCV), and human papillomavirus (HPV) remain significant global health burdens [2-4]. In South Asia, CVD accounts for nearly 35% of deaths, while HBV prevalence is about 6% in Pakistan, 5% in Bangladesh, and 3% in India [5,6]. These overlapping burdens underscore the need to investigate the relationship between oncogenic viral infections and cardiovascular risk in this region.

Chronic viral infection, especially herpes and hepatitis viruses, has been associated with an increased long-term risk of CVD development [7]. Oncoviruses like HPV, HBV, HCV, Epstein-Barr virus (EBV), Kaposi's sarcoma-associated herpesvirus (KSHV), and human T-cell lymphotropic virus type 1 (HTLV-1) play a role in causing at least 12% of human cancer, whose initiation begins by inducing chronic inflammation, cell loss, and proliferation mostly among persons who have weakened immune systems or are less exposed to the healthcare system [8,9].

Myocarditis and dilated cardiomyopathy (DCM) are most often caused by viral infections, such as enteroviruses, parvovirus B19, coxsackievirus B3, human herpesvirus 6, and, more recently, severe acute respiratory syndrome coronavirus 2 (SARS-CoV-2). The disease is both difficult to diagnose and treat, despite advancements in the field of diagnostics. It has been found that certain viruses can enter a state of latency in cardiac tissue and reactivate, causing long-term cardiac damage [10,11]. Viral infections may strain the heart by altering vascular resistance, congenital malformations, or by invading the heart muscle to produce myocarditis [12].

The prevalence of HCV infection among the population is high due to the presence of metabolic disorders and cardiovascular changes. It is essential to determine and understand whether the connection between HCV infection and certain cardiometabolic disorders is coincidental or based on premises of pathogenetic mechanisms, to comprehend the broader scope of the disease [13]. Over 150 studies have identified chronic infections, including *Helicobacter pylori*, *Chlamydia pneumoniae*, and cytomegalovirus (CMV), with vascular disease, specifically coronary heart disease. Nevertheless, the precise causal associations are unclear, and future studies, such as larger seroepidemiologic investigations and randomised trials, are required [14]. Recent studies propose that HPV can be an unconventional hazard to heart disease (CVD) by causing vascular harm, inflammation, and obstruction of lipid metabolism. This supports that specific HPV screening and immunisations are possible to prevent CVDs [15]. Other oncogenic viruses have also been linked to cardiovascular injury. HBV has shown inflammatory associations with coronary artery disease, while EBV can cause myocarditis and arrhythmias through immune-mediated injury. KSHV and HTLV-1 may contribute to endothelial dysfunction and inflammatory cardiomyopathy [16-19]. 

This study aims to investigate the potential threats to cardiovascular health posed by oncogenic viral infections, as well as whether such infections can exacerbate cardiovascular risks, initiate chronic vascular pathogenesis, and determine the extent to which these cardiovascular risks contribute to the burden of cardiovascular diseases in the population. By shedding light on this relationship in this way, we aim to develop novel preventive approaches and therapeutics that address individuals with both types of viral infections and cardiovascular diseases.

Rationale

There is an emerging global burden of both CVDs and viral infections, suggesting a necessity to investigate possible common risk factors. Although the influence of viral infections on cancer development has been well established, the same is not true for cardiovascular health. This knowledge gap is especially concerning, given the growing evidence that viral infections contribute to the development of CVDs through processes such as inflammation, vascular endothelial damage, and immune dysregulation. Exploring the potential cardiovascular implications of oncogenic viral infections is crucial for determining whether individuals infected with these viruses have an increased risk of developing cardiovascular disorders. This study was conducted in Pakistan and India to obtain region-specific information on oncogenic viral infections and cardiovascular health.

Furthermore, the coexistence of dual threats in the form of oncogenic viral infections and CVDs may have serious adverse consequences for the population's health, especially in regions affected by both oncogenic viral infections and CVDs. Due to the chronic character of these conditions, the early signs of cardiovascular risk in patients with oncogenic viral infections may serve as a basis for more successful screening and intervention plans. The current research addresses a pressing gap in the existing literature, which will contribute to a greater understanding of the potential mechanisms underlying increased cardiovascular risks associated with viral infections and subsequently add to the complex information necessary for more effective management and preventive care of affected individuals.

Objectives

The objectives of this study were to identify the relationship between oncogenic viruses (including HPV, HBV, HCV, and EBV) and the prevalence of cardiovascular risks among affected persons, determine the possible relationships between the duration and severity of virus infections, as well as having cardiovascular risk factors, based on questionnaire data, and investigate the possibility of whether demographic and clinical features such as age, gender, and the type of viral infection have an impact on the outcome of cardiovascular health in survey data.

## Materials and methods

Study design

The study used a cross-sectional design to investigate the correlation between oncogenic viral infections and cardiovascular risk factors among adults. Although a cross-sectional design cannot establish causation, it enables an in-depth examination of the relationship between viral infections and subclinical cardiovascular symptoms. This study proposes necessary baseline information that can be harnessed in future studies exploring the causal relationship between oncogenic viral pathogenicity and cardiovascular health. This design has facilitated the assessment of potential cardiovascular risk factors associated with oncogenic viral infections, thereby contributing to the understanding of early cardiovascular stress investigations among this population. The outcomes are likely to provide insightful input in the formulation of preventive strategies and health-related interventions to address the consequences of oncogenic viral infections among adults.

Study population

Convenience sampling was used, meaning eligible participants who visited the participating community center and outpatient clinics during the study period were invited to participate. Participants were recruited from three clinics: two in Pakistan (a community-based center in Lahore and an outpatient clinic in Dera Ghazi Khan) and one outpatient clinic in Haryana, India, to ensure representation from diverse South Asian communities, affected by a high rate of both oncogenic viral infections and CADs, enhancing the generalizability of the findings. Table 1 presents the inclusion and exclusion criteria applied for participant selection in this study.

**Table 1:** Inclusion and exclusion criteria HPV: human papillomavirus; HBV: hepatitis B virus; HCV: hepatitis C virus; EBV: Epstein-Barr virus

Criteria Type	Details
Inclusion	Participants must be at least 18 years old.
Inclusion	History of oncogenic infections with viruses such as HPV, HBV, HCV, or EBV, as reported via self-report or medical history
Inclusion	No known history of cardiovascular diseases (e.g., hypertension, heart disease, stroke)
Inclusion	Ability to provide informed consent
Inclusion	Capable of interpreting the structured questionnaire and responding to it either with or without the help of a trained interviewer.
Exclusion	People who were younger than 18 years old
Exclusion	Known history of cardiovascular diseases, including coronary artery disease, arrhythmias, or heart failure
Exclusion	People infected with non-oncogenic viruses, including the common cold or influenza
Exclusion	Pregnant individuals (due to physiological changes in the cardiovascular and immune systems)
Exclusion	Severe cognitive impairments prevent comprehension or questionnaire completion
Exclusion	Currently enrolled in a clinical trial likely to influence study results.

Sample size and technique

To calculate the sample size for the given study, the standard formula for estimating the number of people to be included in the study when the population is not limited was used, as no previous data were available on the true prevalence of oncogenic viral infections and cardiovascular risk factors among the planned population. The formula employed was:

\[n = \frac{Z^2 \cdot p (1 - p)}{d^2}\]

Where the standard score is Z at the desired level of confidence (1.96 in case of 95% confidence), p is the approximated proportion (0.50 selected to result in the maximum sample size possible), and d is the error margin (it is set at 0.05) [20]. This standard formula was used because the study design was cross-sectional, aiming to estimate the prevalence (proportion) of oncogenic viral infections and their cardiovascular risk within a population. It is the most widely accepted approach for determining sample size in prevalence studies where the primary outcome is a proportion. Other formulas, such as those for cohort or case-control studies, were not applicable since the objective was not to compare groups or assess incidence over time.

According to these parameters, the estimated sample size required was 384 participants. However, the final analysis included 203 participants due to practical limitations, such as restricted participant availability, varying response rates, and logistical constraints in multi-country data collection. While this may slightly reduce statistical power, efforts were made to recruit from diverse locations to enhance representativeness. This method made it easy to collect data on the available participants; however, it also introduced the risk of sampling bias. To reduce this, attempts were made to recruit participants from diverse locations, thereby ensuring a diverse socioeconomic and educational background, which contributed to the increased representativeness of the sample.

Data collection

This study utilised structured questionnaires as a data-gathering tool, which had three major sections: demographic data, the presence or absence of prior viral infections, and cardiovascular risk potential factors. These questionnaires were not modified in terms of their original language and cultural adaptations.

Demographic Information

The initial section of the questionnaire was used to collect demographic information to explore possible relationships between individual characteristics and subclinical cardiovascular symptoms. Age, gender, marital status, education level, occupation, and area of residence were given as the variables. The section helped to profile the study population and make comparisons among populations (see Appendix A).

Viral Infection History

One of the components of the questionnaire aimed to assess the history of oncogenic virus infections in participants, including those caused by HPV, HBV, HCV, and EBV. Respondents were questioned about their experience with such viruses, including prior diagnoses and the treatments or interventions they received as a result. This section helped identify the viral status of the study participants and determine whether it is associated with cardiovascular risk factors (see Appendix B).

Kansas City Cardiomyopathy Questionnaire (KCCQ-12)

The KCCQ-12 was also used in the present study to measure the participants' perceived quality of life in terms of heart failure symptoms, comprising 12 items. Dr. John A. Spertus and other associates of the Mid-America Heart Institute developed it in the year 2000 [21]. The KCCQ-12 has four domains: physical limitation, symptom frequency, quality of life, and social restraint. The individual score (0-100) is used, where a higher value indicates a healthier state. Each domain is averaged to obtain a summary score. The KCCQ-12 is a highly consistent test, with a Cronbach's alpha of 0.89 reported. Since it is a self-reported and brief tool, it was used to determine how the symptoms of heart failure affect the daily functioning and overall wellbeing of the person [21]. Formal permission and license (License ID: 13659) to use the KCCQ-12 were obtained from Outcomes Instruments, LLC, for this non-commercial academic study.

INTERHEART Modifiable Risk Score (IHMRS)

This study uses the IHMRS, which was developed by McGorrian et al. in 2010 [22] as a derivative of the original INTERHEART study by Yusuf et al., published in 2004 [23]. It is an instrument that estimates the likelihood of a person having cardiovascular problems based on the modifiable risk factors of the lifestyle, such as smoking, hypertension, diabetes, weight, diet, exercise, stress, and drinking alcohol. The IHMRS has also demonstrated optimal performance in various populations, including South Asian populations, with an area under the receiver operating characteristic curve (AUC) often exceeding 0.75, indicating its utility in distinguishing between individuals with and without cardiovascular risk. IHMRS is an excellent tool for measuring cardiovascular risk, as it is easy and consistent [22]. Permission to use the IHMRS was obtained through direct email correspondence with the original developers, with approval granted for academic use and acknowledgement in all resulting publications.

Procedure

After providing informed consent, participants were invited to participate in the research. Potential participants were approached by trained research personnel, who explained the study objectives, eligibility criteria, and procedures before obtaining consent. The recruitment process was conducted in community centres and at outpatient clinics in Pakistan and India, allowing for the participation of participants from two South Asian nations. Data collection took place between May 2025 and August 2025, spanning a total of four months. Respondents could complete the questionnaire independently or seek assistance from well-trained research personnel, depending on their individual preferences or level of expertise. All interviewers underwent standardized training to ensure consistent administration of the questionnaire. On average, participants completed the survey within 20-30 minutes, and clarifications were provided when necessary without influencing responses. The answers provided by the respondents were submitted anonymously, without any personal information that could be linked to their responses, to ensure confidentiality.

The IHMRS was employed to enable the participants to self-report about many lifestyle issues, such as smoking, physical activity, alcohol intake, and history of viral infection. In addition, a history of viral infections was established through a sequence of specialised questions regarding diagnosis, treatment, and symptom severity. The symptoms of CVD were not directly evaluated; instead, they were determined based on questions referring to lifestyle and the condition of the viral infection. This approach was designed to determine the extent to which these factors influence cardiovascular risks among individuals with oncogenic viral infections. This ensured the data sample was representative, neutral, and ethically controlled through the inclusion of adults from diverse educational and socioeconomic backgrounds.

Statistical analysis

The statistical analyses were done in IBM SPSS Statistics for Windows, version 26 (IBM Corp., Armonk, New York, United States). The data were summarised by describing the demographic characteristics of the participants in terms of frequencies and percentages. Data were examined for normality using the Shapiro-Wilk test prior to conducting non-parametric analyses. The Spearman ranking order correlation coefficients were used because the data are not parametric, and hence the objective of analysing the associations between study variables. Because the data were not normally distributed, we employed the Mann-Whitney U test to compare the distribution of the two variables related to viral infection and health scores by gender, using the information provided by the participants, who were male and female. Comparisons among multiple groups incorporated the Kruskal-Wallis H formula to determine the differences between the KCCQ and IHMRS scores for types of viral infections and the current infection status of the virus. The predictive values of the KCCQ scores were determined by regression analysis, using variables such as IHMRS and viral infection, while also considering solicited possible confounders. These variables were identified as significant predictors in the regression model, which examined, among other factors, the type of viral infection, medical treatment, symptom severity, and vaccination. Finally, chi-square tests were also performed to explore the associations between viral diagnosis, symptom severity, vaccination history, and the passage of time since the viral Diagnosis. All tests were conducted using a p-value of less than 0.05 to identify the factors that influenced cardiovascular risk and symptom severity in participants with viral infections.

Ethical considerations

The study was conducted in accordance with all ethical requirements for research involving human subjects, thereby demonstrating sound moral principles. The study was approved by the Institutional Review Board of the CMH Lahore Medical College and the Institute of Dentistry (approval number: IRB/CMH/2025/034B). Confidentiality was maintained throughout the study. Personal identifiers were not recorded, and all data were used solely for research purposes. Incomplete responses were handled using the pairwise deletion method to maximize the use of available data. Participants with less than 20% survey completion were excluded from the analysis to ensure validity and reliability. 

## Results

Table 2 presents general demographic characteristics along with viral infection-related variables; cardiovascular risk and health outcomes are summarized separately in subsequent tables. Regarding the age distribution, the highest proportion is categorised among respondents aged 36-45 years, with 60 respondents (30%), followed by those aged 46-55 years, with 45 respondents (22%). Gender-wise, 93 participants (46%) were male and 110 (54%) were female. In terms of marital status, most individuals were either married (74 participants, 36%) or divorced (73, 36%), whereas 27 participants (13%) were single and 29 (14%) had been widowed. The educational level was diverse, with 61 (30%) participants having secondary education, 59 (29%) possessing intermediate/F.A./FSc, and 44 (22%) having primary education. Occupationally, 93 (46%) were retired, 67 (33%) unemployed, 33 (16%) employed, and 10 (5%) students. Regarding residence, 149 (73.2%) respondents lived in urban settings, and 54 (27.8%) respondents lived in rural settings. Regarding viral infections, HBV was the most common, with 62 participants (31%) diagnosed, compared to HCV (n=54, 26%) and HPV (n=9, 4%). The vast majority of participants (n=75, 37%) reported being diagnosed with viral infections more than six years ago. Regarding the existing condition of such infections, it was determined that 82 participants (40%) reported that these infections had been treated and cured, while 53 (26%) claimed to have long-term, stable infections. Treatment data demonstrated that 99 patients (49%) were placed under supportive care, and 64 (31%) were not treated. Regarding the severity of symptoms, 95 (47%) participants experienced severe symptoms that required hospitalisation, while 35 (17%) were asymptomatic. Lastly, the vaccination rate revealed that 105 participants (52%) had received the HPV vaccine, compared to 73 participants (36%) who had received the HBV, and 20 participants (10%) who had received both.

**Table 2:** Demographic characteristics of participants (N = 203) HPV: human papillomavirus; HCV: hepatitis C virus; EBV: Epstein-Barr virus; HTLV: human T-cell lymphotropic virus

Variable	Frequency	Percentage
Age	-	-
18–25 years	6	2.96
26–35 years	20	9.85
36–45 years	60	29.56
46–55 years	45	22.17
56–60 years	24	11.82
60 years & above	48	23.65
Gender	-	–
Male	93	45.81
Female	110	54.19
Marital status	-	–
Single	27	13.30
Married	74	36.45
Divorced	73	35.96
Widowed	29	14.29
Educational level	-	–
No formal education	13	6.40
Primary	44	21.67
Secondary	61	30.05
Intermediate/F.A/FSc	59	29.06
Bachelor's degree	22	10.84
Master's degree	4	1.97
Occupation	-	–
Student	10	4.93
Employed	33	16.26
Unemployed	67	33.00
retired	93	45.81
Residence area	-	–
Urban	149	73.40
Rural	54	26.60
Diagnosis of viral infections (HPV, HBV, HCV, EBV, HTLV)	-	–
Human papillomavirus (HPV)	9	4.43
Hepatitis B virus (HBV)	62	30.54
Hepatitis C virus (HCV)	54	26.60
Epstein-Barr virus (EBV)	24	11.82
Human T-Lymphotropic Virus 1 (HTLV-1)	18	8.87
Other viral infection	36	17.73
Time of diagnosis	-	–
Within the last 1 year	12	5.91
1-3 years	31	15.27
4-6 years	67	33.00
More than 6 years ago	75	36.95
I do not remember	18	8.87
Current status of viral infection(s)	-	–
Active infection (currently under treatment)	34	16.75
Treated and resolved	82	40.39
Chronic but stable	53	26.11
I am not sure	34	16.75
Received medical treatment for viral infection(s)	-	–
Yes, antiviral medication	40	19.70
Yes, supportive care (e.g., fluids, rest)	99	48.77
No treatment was given	64	31.53
Severity of symptoms during infection	-	–
No symptoms (asymptomatic)	35	17.24
Mild symptoms (manageable at home)	28	13.79
Moderate symptoms (needed regular follow-up)	40	19.70
Severe symptoms (required hospitalisation)	95	46.80
I do not remember	5	2.46
Vaccination received	-	–
HPV Vaccine (Gardasil or Cervarix)	105	51.72
Hepatitis B Vaccine	73	35.96
I have received both vaccines	20	9.85
I have not received any of these	5	2.46

Table 3 illustrates significant relationships between various variables related to viral infections and cardiovascular health. Of particular note, the viral infection diagnosis is measured with moderate positive correlations regarding the current state of viral infections (ρ = 0.243, p < 0.01) and vaccination status (ρ = 0.267, p < 0.01). The time of diagnosis also has a negative relationship to the severity of symptoms during infection (ρ = -0.182, p < 0.05), and the longer the diagnosis, the less severe the symptoms of the infection. The KCCQ and IHMRS demonstrate a strong positive connection (r = 0.298, p < 0.01), indicating that individuals with better scores on cardiovascular disease risk, as measured by its short-term risk, will have higher scores on the KCCQ. These results highlight the possible correlations between viral infections and cardiovascular risk factors.

**Table 3:** Spearman's Correlations Among Study Variables (N = 203) Note. Values are Spearman's rank-order correlation coefficients (ρ); p <0.05 (*); p <0.01 (**), N = 203; All percentages used in descriptive analysis are based on valid responses (i.e., excluding missing data) to ensure consistency across variables.

Variables	1	2	3	4	5	6	7	8
Diagnosis of viral infections (HPV, HBV, HCV, EBV, HTLV)	-	0.184^*^	0.215^*^	0.198^*^	–0.176^*^	0.162^*^	-0.208^*^	0.267^**^
Time of Diagnosis	-	-	–0.201^*^	–0.156^*^	–0.145^*^	–0.189^*^	–0.153^*^	0.172^*^
Current status of viral infection(s)	-	-	-	0.183^*^	0.161^*^	0.168^*^	0.219^*^	0.243^**^
Received medical treatment for viral infection(s)	-	-	-	-	–0.174^*^	–0.155^*^	0.172^*^	–0.147^*^
Severity of symptoms during infection	-	-	-	-	-	–0.182^*^	–0.158^*^	0.163^*^
Vaccination received	-	-	-	-	-	-	0.184^*^	–0.165^*^
Kansas City Cardiomyopathy Questionnaire (KCCQ)	-	-	-	-	-	-	-	0.298^**^
INTERHEART Risk Score Questionnaire (IHMRS)	-	-	-	-	-	-	-	-

Table 4 presents the findings of the Mann-Whitney U test comparing male and female patients on all viral infection, vaccination, and questionnaire variables. Gender differences were noted to be significant among some variables. Males had a significantly lower mean rank in the diagnosis of a viral infection (p = .017) and the severity of symptoms in the case of infection (p = .013), suggesting that females reported greater rates of diagnosis and severity of symptoms. Males similarly showed significantly higher average ranks attributed to medical treatment (p = 0.037) and the state of current infection (p = 0.024), indicating a greater likelihood of reporting treatment and showing a currently stable infection status. The KCCQ and IHMRS were also found to be significantly different between genders, with female patients exhibiting higher mean ranks in both of them ( 2.42, p = 0.016, and 2.06, p = 0.040, respectively), pointing to higher health outcomes in female participants. The comparison between vaccination and gender did not reveal a substantial interaction (p = 0.064), indicating that female and male participants had similar vaccination rates.

**Table 4:** Comparison of viral infection, vaccination, and health questionnaire scores between genders (N = 203) Note. N = 203 (males = 93, 46%; females = 110, 54%); Mann–Whitney U test was used for all comparisons; p values marked with * indicate statistical significance at p <0.05. HPV: human papillomavirus; HCV: hepatitis C virus; EBV: Epstein-Barr virus; HTLV: human T-cell lymphotropic virus

Variable	Gender	n (%)	Mean Rank	Sum of Ranks	U	W	Z	P
Diagnosis of viral infections (HPV, HCV, EBV, HTLV)	Male	93 (45.81)	96.12	8949	4471	8949	-2.38	0.017
	Female	110 (54.19)	106.44	11787	-	-	-	-
Time of diagnosis	Male	93 (45.81)	104.85	9741	4663	9741	-1.9	0.057
	Female	110 (54.19)	99.06	10995	-	-	-	-
Current status of viral infection(s)	Male	93 (45.81)	97.54	9071	4515	9071	-2.25	0.024
	Female	110 (54.19)	105.23	11665	-	-	-	-
Received medical treatment for viral infection(s)	Male	93 (45.81)	107.18	9967.5	4612.5	9967.5	-1.85	0.064
	Female	110 (54.19)	97.34	10768.5	-	-	-	-
Severity of symptoms during infection	Male	93 (45.81)	97.23	9042	4620	9042	-2.08	0.04
	Female	110 (54.19)	105.62	11694	-	-	-	-
Vaccination received	Male	93 (45.81)	101.23	9414	4890	9414	-1.9	0.057
	Female	110 (54.19)	102.84	11322	-	-	-	-
Kansas City Cardiomyopathy Questionnaire (KCCQ)	Male	93 (45.81)	106.54	9889	4490.5	9022.5	-2.42	0.016
	Female	110 (54.19)	98.82	10825	-	-	-	-
INTERHEART Modifiable Risk Score (IHMRS)	Male	93 (45.81)	98.66	9177.5	4875	9775	-1.85	0.064
	Female	110 (54.19)	105.95	11737.5	-	-	-	-

Table 5 analysis of the mean ranks comparing the total KCCQ and the total IHRSS scores across the three types of viral infection failed to show any significant difference between them (P = .578). Both the scores of KCCQ and IHMRS showed substantial distinction between the two types of viral infection (and it was revealed in 15.21 and 17.84, respectively). Participants with HPV infection (KCCQ: 135.20, IHMRS: 128.15) and other viral infections (KCCQ: 140.80, IHMRS: 145.40) had the highest mean rank scores on both the KCCQ and IHMRS, indicating that these groups experienced better health outcomes than others. Conversely, those with HTLV-1 reported the lowest scores on the outcomes of their health (KCCQ: 75.28, IHMRS: 78.96). The rates of HBV and HCV infection were also lower in KCCQ, as well as IHMRS scores, indicating moderate health outcomes. These results demonstrate that the type of viral infection can impact the overall health of the cardiovascular system and the risk scores, with some types of infection being associated with less favourable outcomes.

**Table 5:** Comparison of mean ranks of total KCCQ and total IHMRS scores by viral infection type (N = 203) Values are mean ranks from Kruskal–Wallis H tests; Overall test statistics are reported in the bottom row for each dependent variable: Total KCCQ (χ²(5) = 15.21, p <0.001**) and Total IHMRS (χ²(5) = 17.84, p <0.001**); Significance levels: p <0.01**. KCCQ: Kansas City Cardiomyopathy Questionnaire; IHMRS: INTERHEART Modifiable Risk Score

Viral Infection Type	Frequency (Percentage)	Total KCCQ Mean Rank	Total IHMRS Mean Rank
Human Papillomavirus (HPV)	8 (4%)	135.20	128.15
Hepatitis B Virus (HBV)	78 (38%)	112.45	115.38
Hepatitis C Virus (HCV)	56 (28%)	97.30	99.22
Epstein-Barr Virus (EBV)	36 (18%)	86.44	89.57
HTLV-1 (Human T-Lymphotropic Virus 1)	20 (10%)	75.28	78.96
Other viral infection	5 (2%)	140.80	145.40
(χ², df = 5)	-	15.21	17.84
p-value	-	<0.001^**^	<0.001^**^

Table 6 presents the findings of the Kruskal-Wallis test, which measures the average ranks of the total KCCQ scores and the total IHMRS scores according to the current viral infection state (n = 203). There were significant differences in the scores of KCCQ and IHMRS across the four groups based on the status of viral infection (χ²(3) = 14.82, p < 0.001; χ²(3) = 17.04, p < 0.001, respectively). Individuals under treatment for active infections had the highest mean ranks of 125.42 and 130.36 on the KCCQ and IHMRS, respectively, reflecting relatively improved cardiovascular health compared to other categories. Patients whose infections were successfully treated and resolved had moderate mean ranks (KCCQ: 118.33, IHMRS: 122.41). Conversely, those individuals who had chronically stabilised infections showed lower values for both KCCQ (92.18) and IHMRS (96.07) scores, implying poorer cardiovascular health. The mean ranks, which were the lowest, belonged to those who were uncertain about the infection and its reaction (KCCQ: 78.56, IHMRS: 74.12), indicating the poorest health outcomes in this category. These findings demonstrate that the status of viral infection plays a significant role in the health and risk of cardiovascular disease, and inactive infection can be linked to improved health.

**Table 6:** Kruskal–Wallis test results for differences in KCCQ and IHMRS scores by current status of viral infection (N = 203) Values are mean ranks from Kruskal–Wallis H tests; Overall test statistics are reported in the bottom row for each dependent variable: Total KCCQ (χ²(3) = 14.82, p <0.001**) and Total IHMRS (χ²(3) = 17.04, p <0.001**); Significance levels: p <0.01**. KCCQ: Kansas City Cardiomyopathy Questionnaire; IHMRS: INTERHEART Risk Score Questionnaire

Current Status of Viral Infection	Frequency (Percentage)	Total KCCQ Mean Rank	Total IHMRS Mean Rank
Active infection (currently under treatment)	34 (17%)	125.42	130.36
Treated and resolved	82 (40%)	118.33	122.41
Chronic but stable	53 (26%)	92.18	96.07
I am not sure	34 (17%)	78.56	74.12
(χ², df = 3)	-	14.82	17.04
p-value	-	<0.001^**^	<0.001^**^

Table 7 presents a multiple regression analysis of predictors of total KCCQ scores, based on variables reflecting viral infection and the IHMRS. The results demonstrate that KCCQ scores are heavily dependent on several factors, with the IHMRS proving to be the most powerful determinant (β = 0.322, p < 0.001). Notably, the higher the risk score, the worse the cardiovascular health status. There are also significant differences in viral infection variables, as the current status of infection (β = 0.152, p = 0.003) and time of diagnosis (β = -0.156, p = 0.004) were predictors of poorer outcomes, with earlier diagnoses and active infections being associated with poorer outcomes. Moreover, medical treatment (β = 0.118, p = 0.002), vaccination (β = 0.108, p = 0.002), and treatment and prevention have been shown to have a positive impact on KCCQ scores, implying that the positive effects of treatment and vaccination can affect cardiovascular health positively in patients with viral infections.

**Table 7:** Multiple regression predicting total KCCQ from viral infection–related variables and IHMRS (N = 203) All predictors were entered; p <0.01**, p <0.001**. KCCQ: Kansas City Cardiomyopathy Questionnaire; IHMRS: INTERHEART Modifiable Risk Score; HPV: human papillomavirus; HBV: hepatitis B virus; HCV: hepatitis C virus; EBV: Epstein-Barr virus; HTLV: human T-cell lymphotropic virus; B: unstandardized regression coefficient; SE: standard error; β: standardised regression coefficient; CI: confidence interval; LL: lower limit; UL: upper limit;

Predictor	B	SE B	β	t	p	95% CI UL	LL
Constant	18.652	3.782	—	4.932	<0.001^**^	11.20	26.10
Diagnosis of viral infections (HPV, HBV, HCV, EBV, HTLV)	-0.842	0.268	-0.167	-3.141	0.002^**^	-1.37	-0.31
Time of Diagnosis	-0.658	0.226	-0.156	-2.915	0.004^**^	-1.10	-0.21
Current status of viral infection(s)	0.721	0.241	0.152	2.991	0.003^**^	0.25	1.19
Received medical treatment for viral infection(s)	0.955	0.305	0.118	3.131	0.002^**^	0.35	1.55
Severity of symptoms during infection	-0.532	0.180	-0.121	-2.956	0.003^**^	-0.89	-0.18
Vaccination received	0.814	0.256	0.108	3.180	0.002^**^	0.31	1.32
INTERHEART Risk Score Questionnaire (IHMRS)	0.562	0.095	0.322	5.916	<0.001^**^	0.37	0.75

Table 8 shows the cross-tabulation of viral diagnosis and symptom severity during infection. A strong relationship existed between the type of viral infection and the severity of the symptoms (χ²(20) = 156.4, p < 0.001). In the case of HPV, most people did not experience symptoms, with seven (78%) having no symptoms and zero persons having severe symptoms. Conversely, HBV showed a high proportion of patients who were severely attacked and admitted to hospitals, where 46 (74%) of the 62 cases had experienced severe symptoms. HCV presented with a proportional pattern of severe symptoms, with 31 (57%) of the 54 participants exhibiting moderate signs that required frequent monitoring, and two (4%) participants reporting severe signs. The incidence of people with severe symptoms in EBV was also high, with seven (29%) of the 24 participants with EBV reporting severe symptoms and nine (37%) showing no symptoms. HTLV-1 was primarily linked to severe symptoms, where 15 (83%) of 18 participants reported severe symptoms, whereas only two (11%) reported no symptoms. Finally, other viral infections demonstrated a larger prevalence of severe symptoms, as 25 (69%) of 36 respondents indicated severe symptoms. These results suggest that the symptoms differ in intensity across viral infection types, with certain ones (e.g., HBV, HTLV-1) causing more severe effects, whereas others (e.g., HPV) may cause less dramatic consequences.

**Table 8:** Cross-tabulation of viral diagnosis and severity of symptoms during infection (N = 203) χ²(20) = 156.4, p = <0.001**, indicating a statistically significant association between type of viral infection and severity of symptoms

Type of Virus	No symptoms (asymptomatic)	Mild symptoms (manageable at home)	Moderate symptoms (needed regular follow-up)	Severe symptoms (required hospitalisation)	I do not remember	Total	x^2^ (20)	p
Human Papillomavirus (HPV)	7 (78%)	2 (22%)	0 (0%)	0 (0%)	0 (0%)	9	-	-
Hepatitis B Virus (HBV)	0 (0%)	13 (21%)	3 (5%)	46 (74%)	0 (0%)	62	-	-
Hepatitis C Virus (HCV)	14 (26%)	3 (6%)	31 (57%)	2 (4%)	4 (7%)	54	-	-
Epstein–Barr Virus (EBV)	9 (37%)	5 (21%)	2 (8%)	7 (29%)	1 (4%)	24	-	-
HTLV-1 (Human T-Lymphotropic Virus 1)	2 (11%)	1 (6%)	0 (0%)	15 (83%)	0 (0%)	18	-	-
Other viral infection	3 (8%)	4 (11%)	4 (11%)	25 (69%)	0 (0%)	36	-	-
Total	35 (17%)	28 (14%)	40 (20%)	95 (47%)	5 (2%)	203	156.4	<0.001^**^

Table 9 presents the cross-tabulation of vaccination status and the time since diagnosis of viral infection, demonstrating statistically significant differences (χ² (12) = 24.5, p = 0.016). The majority of people who received the HPV vaccine (Gardasil or Cervarix) were already diagnosed more than four years ago, with 38 (36%) diagnosed between four to six years ago, and the other 38 (36%) diagnosed more than six years ago. A similar trend was observed in the HBV vaccine, with 30 (41%) of the 73 respondents reporting health conditions that had occurred over six years prior. The most frequent diagnostic years for those receiving both vaccines were four to six years ago and more than six years ago. The three individuals who did not take any vaccinations were evenly distributed, with one (20%) in each of the four time categories. The significant association suggests that individuals who received HPV or HBV vaccines were more likely to have been diagnosed several years earlier, indicating that vaccination was typically administered post diagnosis.

**Table 9:** Relationship between vaccination status (HPV, HBV, both, or none) and time since viral diagnosis (N = 203) The chi-square test indicates a significant association between vaccination status and time since viral Diagnosis; x2(12) = 24.5, p 0.01** HPV: human papillomavirus; HBV: hepatitis B virus

Vaccination Status	Within the last 1 year, n (%)	1–3 years, n (%)	4–6 years, n (%)	More than 6 years ago, n(%)	I do not remember, n (%)	Total	x^2^(12)	p
HPV Vaccine (Gardasil or Cervarix)	3 (3%)	15 (14%)	38 (36%)	38 (36%)	7 (7%)	105	-	-
Hepatitis B Vaccine	6 (8%)	10 (14%)	20 (27%)	30 (41%)	7 (10%)	73	-	-
Both Vaccines	2 (10%)	3 (15%)	6 (30%)	6 (30%)	3 (15%)	20	-	-
No Vaccines	1 (20%)	1(20%)	1 (20%)	1 (20%)	1 (20%)	5	-	-
Total	12 (6%)	31 (14%)	67 (33%)	75 (37%)	18 (9%)	203	24.5	0.016^**^

## Discussion

The purpose of this study was to investigate the cardiovascular risks associated with oncogenic viral infections, including HPV, HBV, HCV, EBV, and HTLV-1, and their potential contribution to the complications of persistent vascular damage and the cardiovascular disease (CVD) burden. Our findings suggest that the timing of viral diagnosis plays a vital role in patient outcomes. Specifically, we observed a positive relationship between the time elapsed from diagnosis to reporting and the incidence of viral infections. This observation supports previous research suggesting that better control over the diagnostic interval can influence the risk ratio in several health outcomes [24]. In our study, more recent diagnoses were associated with a higher likelihood of current infection but, paradoxically, also with less severe symptoms. This pattern may reflect the acute stage of disease or the benefits of timely treatment, which aligns with previous studies showing that early management improves prognosis and reduces adverse outcomes [25-28]. Furthermore, individuals newly diagnosed had lower vaccination rates, highlighting a potential area for public health intervention to reduce infection severity through timely immunization [29].

Acute infections in our study were generally associated with milder symptoms, likely reflecting the early stage of viral activity and immune response. This observation aligns with prior evidence showing that symptom severity correlates with inflammation, suggesting that higher inflammatory activity is associated with more severe manifestations [30]. Patients with active infections were more likely to receive treatment, consistent with the notion that the presentation of symptoms often prompts clinical intervention. However, we also observed that the most severe infections were less likely to receive additional individualized care, likely due to the standardization of treatment protocols for critically ill patients, where management becomes increasingly protocol-based rather than tailored to evolving symptoms [31].

Vaccination appeared to have a protective effect, with improved KCCQ scores suggesting better cardiomyopathy outcomes. This supports prior studies indicating that immunization reduces the risk of hospitalization and improves cardiovascular outcomes, particularly in vulnerable populations [32]. Interestingly, while heart function improved, cardiovascular risk, as measured by IHMRS, did not necessarily decrease, highlighting the complex interplay between infection, cardiac performance, and other risk factors, such as blood pressure, metabolic disturbances, and heart rate, which remain independent predictors of cardiovascular morbidity and mortality [33]. These findings underscore the importance of integrating preventive measures, such as vaccination, with comprehensive management of underlying cardiovascular risk.

Gender differences emerged as another important factor in viral infection and cardiovascular health. The female population was more frequently diagnosed with viral infections, possibly due to stronger immune responses and greater cytokine activity. In contrast, male participants reported a higher history of active disease and were more likely to seek medical treatment [34-37]. We have concluded that the symptoms of viral infections were more profound in the female participants, and this agrees with other reports that show gender-based disparities in viral load and the disease outcome, with a higher viral load and more severe illnesses reported in women [38]. Our findings revealed that the difference between male and female participants receiving a vaccination was not statistically significant, indicating a tendency but no considerable value. This result, however, does not align with a systematic review, which found a much lower willingness to be vaccinated, especially among women and specifically among healthcare workers, indicating that some gender-based determinants may contribute to vaccination uptake [39]. Interestingly, female participants in our study had higher KCCQ scores, despite generally having a higher cardiovascular risk, emphasizing that gender-related differences in HRQoL and cardiovascular health are complex and likely influenced by both biological and psychosocial factors [40,41]. These observations suggest a need for gender-tailored strategies in both screening and treatment to optimize outcomes.

In our research, the infected participants with HTLV-1 ranked lowest in the KCCQ and the IHMRS, indicating worse cardiovascular health. This agrees with other studies showing that HTLV-1 is associated with worse health outcomes, especially in cardiovascular and related diseases [42]. The strong correlation between HTLV-1 and poor cardiovascular health outcomes, as measured by both the KCCQ and IHMRS scoring systems, underscores the need to investigate the broader role of HTLV-1 in contributing to poor cardiovascular health.

Participants with active infections who were receiving treatment demonstrated better cardiovascular health, suggesting that timely medical care can mitigate the adverse effects of disease on the heart. While awareness of infection can sometimes cause stress and reduce perceived quality of life, engagement in treatment may promote proactive health behaviors and improve overall wellbeing [43,44]. In contrast, a history of viral infections, particularly when diagnosed later in life, was associated with poorer cardiovascular outcomes, likely due to the cumulative effects of persistent viral activity, vascular damage, and dysregulation of the renin-angiotensin system [11,45]. These findings underscore the importance of early detection and intervention to prevent long-term cardiovascular complications.

Individuals with active viral infections who received treatment showed improved cardiovascular outcomes, as reflected by higher KCCQ scores. This aligns with prior research on viral myocarditis, which suggests that timely and effective treatment can positively influence prognosis and symptom management in cardiac patients [10]. Severe infection-related symptoms were associated with poorer cardiovascular health, reinforcing evidence that serious infections leading to hospitalization carry both short-term and long-term cardiovascular risks [46]. Vaccination also appeared to confer protective benefits, contributing to better heart function and potentially reducing hospitalizations, consistent with findings in heart failure populations [32]. Nonetheless, underlying cardiovascular risk factors, including elevated heart rate, blood pressure, and metabolic disturbances, remained significant predictors of adverse outcomes, emphasizing the importance of comprehensive risk management alongside infection control [33].

Patients infected with HTLV-1 exhibited heterogeneous, often moderate-to-severe symptoms, particularly affecting the lower limbs, consistent with previous reports on HTLV-1-associated myelopathy/tropical spastic paraparesis (HAM/TSP), which can substantially impact quality of life [47]. Vaccinated individuals in this group demonstrated improved outcomes, indicating potential benefits of immunization even post-infection. However, prior studies emphasize that vaccination is most effective when administered before viral exposure, highlighting the need for further research to determine optimal timing and long-term efficacy [48].

Limitations

Although this study has provided valuable insights, several limitations must be acknowledged. To start with, the cross-sectional nature of the study limits our ability to determine causal relationships between cardiovascular outcomes and viral infections. Future longitudinal studies are necessary to evaluate the long-term effects of viral infections on cardiovascular health. Second, we have used convenience sampling, which can also be sufficient, although it may introduce bias in the choice of sampling, as participants with certain socioeconomic levels are represented in disproportionate numbers. This may limit the extent to which the results can be generalized to other populations. Additionally, there is a likelihood of a recall bias in self-reports of previous viral infection and cardiovascular symptoms, which was offset to some extent by the utilisation of a structured questionnaire. Lastly, the study did not include clinical measures of cardiovascular well-being, such as blood pressure, cholesterol levels, and echocardiography, which would have provided a more comprehensive physical health picture.

Future directions

Future research should employ a longitudinal design to gain a deeper understanding of the causal relationship between viral oncogenic pathogens and cardiovascular diseases. The general demographic information, combined with clinical cardiovascular factors, can provide further insight into the mechanisms of cardiovascular risk among these populations. Additionally, the potential impacts of vaccination interventions on reducing cardiovascular risks in high-risk populations susceptible to viral infections will need to be researched further. It is possible to conduct studies of the effects of lifestyle components (diet and physical activity) on the mitigation of cardiovascular risk in individuals with chronic viral infections, as well.

## Conclusions

The present study notes a close relationship between cardiovascular risks and oncogenic viruses. The findings show that the continuous viral infection, particularly HBV, HCV, and HTLV-1, causes subclinical cardiovascular manifestations and may predispose individuals to the incidence of CADs. The benefits of early intervention, such as including antiviral therapy and immunization, have suggested a cardiovascular protective effect on such individuals. Given the growing rates of viral infections and CADs, this study emphasizes the urgent need to address these two compounding risks and discusses a strategy that prioritizes early diagnosis, prevention measures, and management.

## Appendices

Appendix A 

**Table 10:** Demographic Information Questionnaire

Items	Responses
Age	-
Gender	-
Marital Status	-
Education	-
Occupation	-
Area of Residence	-

Appendix B 

**Table 11:** Viral Infection History

Items	Responses
Have you ever been diagnosed by a doctor with any of the following viruses?	-
When were you diagnosed with the viral infection(s)?	-
What is the current status of your viral infection(s)?	-
Did you receive medical treatment for the viral infection(s)?	-
What was the severity of your symptoms during the infection?	-
Have you received any of the following vaccines?	-

## References

[1] Deaton C, Froelicher ES, Wu LH, Ho C, Shishani K, Jaarsma T (2011). The global burden of cardiovascular disease. Eur J Cardiovasc Nurs.

[2] (2025). World Health Organization: Cardiovascular diseases (CVDs). https://www.who.int/news-room/fact-sheets/detail/cardiovascular-diseases-(cvds).

[3] (2025). World Health Organization: Global hepatitis report 2024: action for access in low- and middle-income countries. World Health Organization.

[4] (2025). World Health Organization. Cervical cancer. https://www.who.int/news-room/fact-sheets/detail/cervical-cancer.

[5] Joseph P, Kutty VR, Mohan V (2022). Cardiovascular disease, mortality, and their associations with modifiable risk factors in a multi-national South Asia cohort: a PURE substudy. Eur Heart J.

[6] Hogan S, Page A, Dixit S, McBride KA (2023). HBV prevalence in sub-continental countries: a systematic review and meta-analysis. PLoS One.

[7] Yang L, Lu Z, Bian J, Li F, Zou H (2024). Association between chronic viral infection-related hospitalization and risk of cardiovascular disease: a population-based cohort study. J Med Virol.

[8] Gaglia MM, Munger K (2018). More than just oncogenes: mechanisms of tumorigenesis by human viruses. Curr Opin Virol.

[9] Akram N, Imran M, Noreen M, Ahmed F, Atif M, Fatima Z, Bilal Waqar A (2017). Oncogenic role of tumor viruses in humans. Viral Immunol.

[10] Schultheiss HP, Baumeier C, Pietsch H, Bock CT, Poller W, Escher F (2021). Cardiovascular consequences of viral infections: from COVID to other viral diseases. Cardiovasc Res.

[11] Badrinath A, Bhatta S, Kloc A (2022). Persistent viral infections and their role in heart disease. Front Microbiol.

[12] Abelmann WH (1971). Virus and the heart. Circulation.

[13] Petta S, Macaluso FS, Craxì A (2014). Cardiovascular diseases and HCV infection: a simple association or more?. Gut.

[14] Danesh J, Appleby P (1998). Persistent infection and vascular disease: a systematic review. Expert Opin Investig Drugs.

[15] Dutta P, Saha D, Earle M (2024). Unveiling HPV's hidden link: cardiovascular diseases and the viral intrigue. Indian Heart J.

[16] Wijarnpreecha K, Thongprayoon C, Panjawatanan P, Ungprasert P (2016). Hepatitis B virus infection and risk of coronary artery disease: a meta-analysis. Ann Transl Med.

[17] Chen X, Li Y, Deng L (2023). Cardiovascular involvement in Epstein-Barr virus infection. Front Immunol.

[18] Rezaee SA, Cunningham C, Davison AJ, Blackbourn DJ (2006). Kaposi's sarcoma-associated herpesvirus immune modulation: an overview. J Gen Virol.

[19] Izumo S, Umehara F, Osame M (2000). HTLV-I-associated myelopathy. Neuropathology.

[20] Naing L, Nordin RB, Abdul Rahman H, Naing YT (2022). Sample size calculation for prevalence studies using Scalex and ScalaR calculators. BMC Med Res Methodol.

[21] Green CP, Porter CB, Bresnahan DR, Spertus JA (2000). Development and evaluation of the Kansas City Cardiomyopathy Questionnaire: a new health status measure for heart failure. J Am Coll Cardiol.

[22] McGorrian C, Yusuf S, Islam S (2011). Estimating modifiable coronary heart disease risk in multiple regions of the world: the INTERHEART Modifiable Risk Score. Eur Heart J.

[23] Yusuf S, Hawken S, Ôunpuu S (2004). Effect of potentially modifiable risk factors associated with myocardial infarction in 52 countries (the INTERHEART study): case-control study. Lancet.

[24] Seaman SR, Nyberg T, Overton CE, Pascall DJ, Presanis AM, De Angelis D (2022). Adjusting for time of infection or positive test when estimating the risk of a post-infection outcome in an epidemic. Stat Methods Med Res.

[25] Ho GY, Bierman R, Beardsley L, Chang CJ, Burk RD (1998). Natural history of cervicovaginal papillomavirus infection in young women. N Engl J Med.

[26] Millman AJ, Nelson NP, Vellozzi C (2017). Hepatitis C: review of the epidemiology, clinical care, and continued challenges in the direct acting antiviral era. Curr Epidemiol Rep.

[27] Smith-Palmer J, Cerri K, Valentine W (2015). Achieving sustained virologic response in hepatitis C: a systematic review of the clinical, economic and quality of life benefits. BMC Infect Dis.

[28] Mennini FS, Marcellusi A, Robbins Scott S (2021). The impact of direct acting antivirals on hepatitis C virus disease burden and associated costs in four European countries. Liver Int.

[29] Harper DM, Navarro-Alonso JA, Bosch FX (2025). Impact of human papillomavirus vaccines in the reduction of infection, precursor lesions, and cervical cancer: a systematic literature review. Hum Vaccin Immunother.

[30] Haran JP, Suner S, Gardiner F (2012). Correlation of C-reactive protein to severity of symptoms in acute influenza A infection. J Emerg Trauma Shock.

[31] Hranjec T, Sawyer RG (2014). Management of infections in critically ill patients. Surg Infect (Larchmt).

[32] Mohseni H, Kiran A, Khorshidi R, Rahimi K (2017). Influenza vaccination and risk of hospitalization in patients with heart failure: a self-controlled case series study. Eur Heart J.

[33] Tadic M, Cuspidi C, Grassi G (2018). Heart rate as a predictor of cardiovascular risk. Eur J Clin Invest.

[34] Klein SL, Huber S (2009). Sex differences in susceptibility to viral infection. Sex Hormones and Immunity to Infection.

[35] Raboud J, Blitz S, Walmsley S, Thompson C, Rourke SB, Loutfy MR (2010). Effect of gender and calendar year on time to and duration of virologic suppression among antiretroviral-naïve HIV-infected individuals initiating combination antiretroviral therapy. HIV Clin Trials.

[36] McClelland EE, Smith JM (2011). Gender specific differences in the immune response to infection. Arch Immunol Ther Exp (Warsz).

[37] Klein SL (2012). Sex influences immune responses to viruses, and efficacy of prophylaxis and treatments for viral diseases. Bioessays.

[38] Acer Ö, Genç Bahçe Y, Özüdoğru O (2022). Association of viral load with age, gender, disease severity, and death in severe acute respiratory syndrome coronavirus 2 variants. J Med Virol.

[39] Zintel S, Flock C, Arbogast AL, Forster A, von Wagner C, Sieverding M (2022). Gender differences in the intention to get vaccinated against COVID-19: a systematic review and meta-analysis. Z Gesundh Wiss.

[40] Jang JH, Kim DY, Shin SH (2023). Abstract 15228: Impact of gender and left ventricular ejection fraction on health-related quality of life and its change in patients with heart failure. Circulation.

[41] Betai D, Ahmed AS, Saxena P (2024). Gender disparities in cardiovascular disease and their management: a review. Cureus.

[42] Schierhout G, McGregor S, Gessain A (2020). Association between HTLV-1 infection and adverse health outcomes: a systematic review and meta-analysis of epidemiological studies. Lancet Infect Dis.

[43] Dalgard O, Egeland A, Skaug K, Vilimas K, Steen T (2004). Health-related quality of life in active injecting drug users with and without chronic hepatitis C virus infection. Hepatology.

[44] Lowe GD (2001). The relationship between infection, inflammation, and cardiovascular disease: an overview. Ann Periodontol.

[45] Savedchuk S, Raslan R, Nystrom S, Sparks MA (2022). Emerging viral infections and the potential impact on hypertension, cardiovascular disease, and kidney disease. Circ Res.

[46] Sipilä PN, Lindbohm JV, Batty GD (2023). Severe infection and risk of cardiovascular disease: a multicohort study. Circulation.

[47] Dos Santos DN, Sá KN, Queirós FC (2021). Pain, psychoaffective symptoms, and quality of life in human T cell lymphotropic virus type 1 (HTLV-1): a cross-sectional study. J Neurovirol.

[48] Landrum ML, Hullsiek KH, Chun HM (2011). The timing of hepatitis B virus (HBV) immunization relative to human immunodeficiency virus (HIV) diagnosis and the risk of HBV infection following HIV diagnosis. Am J Epidemiol.

